# Barriers to emergency obstetric care services: accounts of survivors of life threatening obstetric complications in Malindi District, Kenya

**DOI:** 10.11694/pamj.supp.2014.17.1.3042

**Published:** 2014-01-18

**Authors:** Elizabeth Echoka, Anselimo Makokha, Dominique Dubourg, Yeri Kombe, Lillian Nyandieka, Jens Byskov

**Affiliations:** 1 Centre for Public Health Research, Kenya Medical Research Institute (KEMRI) Nairobi, Kenya; 2 Department of Food Science Jomo Kenyatta University of Agriculture and Technology, Nairobi, Kenya; 3 Woman and Child Health Research Center, Department of Public Health Institute of Tropical Medicine Nationalestraat 155, 2000, Antwerpen, Belgium; 4 Centre for Health Research and Development, Faculty of Health and Medical Sciences, University of Copenhagen, Thorvaldsensvej 57, Frederiksberg DK 1871, Denmark

**Keywords:** Pregnancy, near miss, EmOC, complication, delay

## Abstract

**Introduction:**

Pregnancy-related mortality and morbidity in most low and middle income countries can be reduced through early recognition of complications, prompt access to care and appropriate medical interventions following obstetric emergencies. We used the three delays framework to explore barriers to emergency obstetric care (EmOC) services by women who experienced life threatening obstetric complications in Malindi District, Kenya.

**Methods:**

A facility-based qualitative study was conducted between November and December 2010. In-depth interviews were conducted with 30 women who experienced obstetric “near miss” at the only public hospital with capacity to provide comprehensive EmOC services in the district.

**Resuls:**

Findings indicate that pregnant women experienced delays in making decision to seek care and in reaching an appropriate care facility. The “first” delay was due to lack of birth preparedness, including failure to identify a health facility for delivery services regardless of antenatal care and to seek care promptly despite recognition of danger signs. The “second” delay was influenced by long distance and inconvenient transport to hospital. These two delays resulted in some women arriving at the hospital too late to save the life of the unborn baby.

**Conclusion:**

Delays in making the decision to seek care when obstetric complications occur, combined with delays in reaching the hospital, contribute to ineffective treatment upon arrival at the hospital. Interventions to reduce maternal mortality and morbidity must adequately consider the pre-hospital challenges faced by pregnant women in order to influence decision making towards addressing the three delays.

## Introduction

Existing evidence suggests that in any population, 1-2 percent of pregnant women will develop life-threatening obstetric conditions during childbirth [1, 2]. If they fail to receive rapid medical interventions, it is likely to result in a maternal death. While complications responsible for most maternal deaths are often unpredictable [3, 4], even in well-nourished and well-educated women who receive sufficient prenatal and delivery care [5], there is evidence that many maternal deaths can be avoided, even in countries with limited resources [6]. That is if women deliver in a setting where skilled attendants can provide emergency obstetric care (EmOC) [3, 7–12].

While sub-Saharan Africa accounts for more than half of the global burden of maternal mortality [13, 14], many women still face challenges in accessing life-saving obstetric interventions [15, 16]. In Kenya, over 50 percent of deliveries are attended to by unskilled persons and/or outside a health facility [17], exposing mothers and babies to poor health outcomes should a complication arise during delivery or postnatal period. The low ratio of EmOC facilities to population size (2.7/500,000) [18], which is below the recommended minimum of 5/500,000 [10], and inequitable access to health services across the country [18, 19] suggests that high maternal mortality in Kenya may result from limited access to life saving interventions for most pregnant women. Maternal mortality is only the tip of an iceberg, the bulk of which is made up of severe morbidity that may lead to long term disability.

The three delays model suggests that pregnancy-related morbidity and mortality stems from delays in (a) making the decision to seek appropriate care, (b) reaching an appropriate facility and (c) receiving adequate and appropriate care once at a health facility [5]. This model provides a valuable theoretical framework for studying the underlying causes of maternal mortality in resource-limited settings. We used this framework to explore the barriers to EmOC services by women who experienced life-threatening obstetric complications or “near miss” in Malindi District, Kenya. According to the district bureau of statistics, Malindi has an estimated maternal mortality ratio of 625 maternal deaths per 100,000 live births compared to 488 deaths per 100,000 live births, nationally [18].

This qualitative paper documents the barriers to EmOC services in Malindi District, from the users’ perspective. It is based on data from a larger study that examined the existence and functionality of EmOC services from a health system perspective in the district [20].

## Methods

### Study setting

This study was conducted in Malindi District (currently Malindi and Magarini Districts) in Kenya. Malindi is located in the southern coastal region and covers an area of 7,792 km2. The total population in the district was 400,514 people in 2009, with an urban/rural distribution of 140,739 and 259,739 persons respectively [21]. The district has a total of 105 public and private health facilities [22], of which 42 (40%) offer maternity services. Among these, there are three hospitals and six health centres, which offer 24 hour/7 days maternity services. The average distance to the nearest health facility is 1 km for urban and 3 km for rural areas. Therefore, the majority of the population is considered to have relatively good access to health facilities. However, the distribution of health facilities is unequal and much of the rural area has poor road infrastructure and limited public transportation, which prevent people from accessing health facilities [23].

The three hospitals provide comprehensive EmOC. Although the coverage of EmOC services (6.2/500,000) is meets the minimum recommended coverage of 5/500,000 rural-urban inequities in geographical distribution of EmOC facilities exist [20]. According to a 2007 population based survey, 84 percent of women in the district deliver at home (REACT project, unpublished).

The study was conducted in the maternity ward of Malindi District Hospital in November-December 2010. The hospital is the only public referral hospital in the district with capacity to provide comprehensive EmOC services (blood transfusion and caesarean section). The hospital has a full time specialist obstetrician/ gynaecologist. In 2010, the hospital recorded a total of 2893 annual deliveries out of 5901 institutional deliveries in the district [20]. The hospital has a 250 bed capacity and a maternity shelter for women with high risk pregnancies. However, the hospital administration notes that the maternity shelter is underutilised as a result of competing demands for women and disruption of household responsibilities as a result of hospital confinement.

### Study population

The participants in this study were female residents of Malindi District, who experienced an obstetric “near miss” or severe acute maternal morbidity and were treated at the district hospital. Near miss is defined as an acute obstetric complication that immediately threatens a woman's survival but does not result directly in her death whether by chance or because of the hospital care she receives during pregnancy, labor or within six weeks after termination of pregnancy or delivery [24]. Since the objective was to understand and give meaning to a social process, rather than quantify and generalize to a wider population, 30 women with were purposively sampled to represent a variety of “near miss” conditions. The sample was deemed adequate to reveal the full range (or nearly the full range) of potentially important insights on pre-hospital and hospital barriers pregnant women with life-threatening obstetric complications experience [25, 26].

The criteria for selecting women who had experienced near miss conditions was based on five categories of obstetric emergencies defined according to disease-specific criteria, based on management and/ or clinical signs and symptoms [24]. These included: haemorrhage at any pregnancy state (leading to transfusion, caesarean section or hysterectomy), hypertensive disorders of pregnancy (eclampsia or severe pre-eclampsia with a minimum diastolic pressure of 110 mmHg), puerperal sepsis (peritonitis, septicemia, offensive vaginal discharge), dystocia resulting from prolonged labor, obstructed labor or malpresentation (leading to ruptured uterus or impending uterine rupture, caesarean section, instrumental delivery or perineal lacerations) and severe anemia (hemoglobin< 6 g/dl).

### Interview guide

An in-depth interview guide was developed based on the three delays framework [5]. The guide focused on knowledge on danger signs during pregnancy, delivery and post partum; antenatal care (ANC) attendance; birth preparedness including choice on where to deliver, identification of health facility for delivery services and saving money; distance and mode of transport to nearest health facility and/ or referral to district hospital; and the woman's experience from onset of complication, arrival at hospital and receiving definitive treatment. Perceptions on quality of care received were also explored. The interview tool was translated from English into Kiswahili, and back-translated to ensure validity of findings.

### Procedure

The women were recruited at the maternity ward on a daily basis over a 4-week period. An attempt was made to interview women with a variety of life-threatening complications from both urban and rural settings. Two female nurse-midwives were trained to recruit the women in the study. Records of all women admitted following an obstetric complication were scrutinised to determine whether the women met the near-miss criteria at admission. If a woman met the criteria, she was approached and informed about the purpose of the study and invited to participate. After obtaining written consent, each woman was interviewed in Kiswahili by a female interviewer at the hospital after the woman had recovered from the life threatening condition. Women who did not speak Kiswahili were interviewed in Kigiriama, which is the local language. The interviews were semi-structured, open ended and included probing to allow women to respond freely. The interviews were audio-recorded and lasted between 30 to 45 minutes. Additionally, a form was used to extract data on age, residence, obstetric complication and intervention performed from the admission records of the participants.

### Data analysis

The qualitative data were analysed thematically using NVivo 9. The recorded interviews were transcribed verbatim and translated from Kiswahili or Kigiriama to English. Transcripts were coded into themes around pertinent issues relating to healthcare seeking and the process of obtaining care. Interview reports were supplemented by information on the type of obstetric condition experienced and management extracted from patient personal case files, theatre and ward daily report books. Attempts were made to identify possible, plausible barriers to the use of EmOC services by the respondents.

### Informed consent and ethical clearance

Ethical approval for this study was granted by the Kenya Medical Research Institute's Ethical Review Committee. Written permission was obtained from the Medical Officer of Health in the district prior to conducting the study. Informed consent was sought from the women and participation was fully voluntary. All information presented in this paper is anonymised.

## Results

### Characteristics of women

All the 30 women interviewed were treated at the hospital, upon arrival in a critical obstetric condition, therefore fulfilling the near miss criteria. Table 1 shows characteristics of the women who were interviewed. The mean age of the women was 25.7 years, ranging from 18 to 39 years. Seventeen women were rural residents. All the women had at least one ANC attendance, with over half having attained the minimum recommended four visits. The most common intervention was caesarean section, indicated by obstructed labour. Severe pre-eclampsia was the second common complication. The first delay was observed for 16 women, the second delay in 8 women, and the third in 6 women. Table 2 shows a summary of residence of and type of delay experienced.

**Table 1:** Characteristics of women admitted at Malindi District Hospital in near miss condition

ID	Age	Area	No. of ANC	Deliverypreference	Complication	Intervention	Outcome of baby
**01**	**30**	**Urban**	**3**	Hospital	OL, FD	CS	Born alive
**02**	**37**	**Urban**	**1**	Hospital	severe PET	MgSO_4_	N/A*
**03**	**30**	**Urban**	**4**	Hospital	severe PET	MgSO_4_	N/A*
**04**	**32**	**Urban**	**4**	Hospital	severe PET	MgSO_4_	N/A*
**05**	**23**	**Urban**	**5**	Hospital	Primi, OL	CS	Born alive
**06**	**18**	**Rural**	**3**	Home	OL, Sepsis	CS, antibiotics	Born dead
**07**	**32**	**Urban**	**1**	Hospital	severe PET	MgSO_4_	N/A*
**08**	**20**	**Urban**	**1**	Hospital	OL	CS	Born alive
**09**	**20**	**Rural**	**3**	Hospital	OL	CS	Born alive
**10**	**19**	**Rural**	**1**	Home	OL, CPD	CS	Born alive
**11**	**22**	**Rural**	**5**	Home	OL, CPD, FD	CS	Born alive
**12**	**23**	**Rural**	**4**	Home	PET, FD	CS	Born alive
**13**	**28**	**Rural**	**5**	Hospital	OL, FD	CS	Born alive
**14**	**23**	**Rural**	**1**	Home	APH, severe PET	BT, MgSO_4_	Born dead
**15**	**24**	**Rural**	**4**	Home	OL, CPD	CS	Born alive
**16**	**39**	**Rural**	**3**	Hospital	Severe PET	MgSO_4_	N/A*
**17**	**32**	**Rural**	**2**	Hospital	Ruptured uterus	CS	Born alive
**18**	**18**	**Rural**	**1**	Home	OL, CPD, FD	CS	Born alive
**19**	**17**	**Urban**	**5**	Hospital	Severe eclampsia, FD	CS	Born dead
**20**	**23**	**Rural**	**3**	Hospital	OL, FD	CS	Born alive
**21**	**25**	**Urban**	**5**	Hospital	OL, FD	CS	Born alive
**22**	**31**	**Urban**	**2**	Hospital	severe PET	MgSO_4_	N/A*
**23**	**29**	**Rural**	**2**	Home	OL, FD	CS	Born dead
**24**	**23**	**Rural**	**4**	Home	severe PET	MgSO_4_	N/A*
**25**	**26**	**Urban**	**5**	Hospital	OL	CS	Born alive
**26**	**18**	**Rural**	**1**	Home	OL	CS	Born alive
**27**	**26**	**Rural**	**1**	Home	severe PET	MgSO_4_	N/A*
**28**	**37**	**Urban**	**4**	Hospital	severe PET	MgSO_4_	Born alive
**29**	**18**	**Rural**	**4**	Home	OL, fully dilated	CS	Born alive
**30**	**29**	**Urban**	**5**	Hospital	OL, severe PET	CS	Born alive

APH (ante partum haemorrhage); CPD (cephalo pelvic disproportion); PPH (postpartum haemorrhage); PS (puerperal sepsis);PP (placenta praevia); FD (foetal distress); PET (Pre-eclampsia); OL (obstructed labor); CS (Caesarean section); MgSO_4_ (magnessium sulphate).

* N/A-condition managed, awaiting delivery

**Table 2:** Distribution of women who experienced near miss by type of delay and residence

Type of delay	Residence		
	Rural	Urban	
**First: Delays in making decision to seek care**	9	7	16
**Second: Delays in reaching care facility**	6	2	8
**Third: Delays in receiving care at facility**	2	4	6
**Total**	17	13	30

### Delays in deciding to seek care

Knowledge of danger signs during pregnancy, delivery and post partum is considered a first step towards initiating appropriate and timely care seeking in the event of an obstetric emergency. A majority of the women mentioned at least two of the following danger signs during pregnancy; heavy vaginal bleeding before the expected delivery date, unpleasant vaginal discharge, water breaking before the due date, abnormal presentation, abdominal pains and dizziness. Common danger signs during child birth were typically described in respondent wording as pushing without the baby coming out, abnormal presentation, the birth canal being too small or failing to open, high blood pressure and too much bleeding. Heavy bleeding was commonly mentioned as a danger sign after delivery.

The women recollected their personal experiences during the near miss episode. It is interesting to note that some women perceived the danger signs to be normal during pregnancy and would not seek medical care immediately. A 19 year old woman who underwent a caesarean section delivery arising from obstructed labor (OL) and cephalo pelvic disproportion (CPD) reported severe pains and only went to hospital because she “was so tired and could not withstand the pain anymore” after several hours of labor. Perceived severity of the illness also seemed to influence decision to seek care. For example, a woman who underwent blood transfusion following severe pre-eclampsia (PET) and ante-partum haemorrhage (APH), but lost the baby described her ordeal:

*“My expected delivery date had not reached. I started having abdominal pains, feeling of fatigued and dizzy in the morning. I dismissed it as normal problems related to pregnancy and would pass. By noon, the pain became severe and I started bleeding heavily. I cannot say much about what happened afterwards because I passed out. My mother in law and other women carried me on a stretcher and brought me to Madunguni Dispensary”* (23 year-old rural resident with APH, severe PET)

Pregnant women are advised to attend ANC and make certain preparations prior to birth. This includes identification of health facility to deliver. All the women interviewed had attended ANC at least once. A majority of the women had attained the recommended four visits, a marker of birth preparedness. However, some still preferred to deliver at home, perceiving the pregnancy as normal often based on previous uncomplicated pregnancies, but ignoring the fact that most complications occur suddenly and are unpredictable. A woman who developed severe PET and ante-partum haemorrhage narrated.

*“Yes I attended ANC four times...I had planned to deliver at home, the same way I did with my two children. I did not have any problem through out this pregnancy. When I started feeling dizzy, I knew it would pass”*. (23 year-old rural resident with APH and severe PET)

The women also narrated how lack of money influenced choice of place of delivery. In some instances, the women had already made advance arrangements to deliver at home with the help of a TBA to cut on costs, as illustrated by the statement below:

*“I had planned to deliver at home with the help of a TBA, so as to reduce on the expenses.When the pain started, I called the TBA, she examined me and advised me to go to the dispensary. At the dispensary, the doctor informed me that they could not assist me because the baby was too big and referred me to Malindi. I came in a Bajaj from Gede to Malindi. We paid KSh 700”*. (22 year-old rural resident with obstructed labor and CPD)

### Delays in reaching care facility

Delays in reaching an appropriate obstetric facility once the decision to seek care has been made constitute the second delay. A majority of the participants from the rural areas first sought care in a dispensary or health centre, from where they were referred to the district hospital. This often meant that women had to organise their own public transport because the ambulances from the district hospital were often unavailable. Poor road infrastructure and transportation contributed to the second delay, particularly for rural women. This is exemplified by narrations of several women.

*“I was first brought to Madunguni dispensary on a stretcher. We had to cross the river. I could not get into the boat because it was too small. They had to carry me on a stretcher and walk across the river because I had passed out...the doctor examined me and immediately called for the ambulance”*. (23 year-old rural resident with APH, severe PET)

*“I was brought to Gongoni health center in a Bajaj (three wheeled cabin cycle). It was at night and I was in so much pain...they called for an ambulance, but were informed that it had gone to pick another patient. At 2pm, the ambulance arrived. We arrived here at around 4pm”* (18 year-old rural resident with obstructed labor and bandl's ring)

*“I started having pains in the morning. I called the TBA to accompany me to the health centre, where I had planned to deliver. When we arrived at around 8 am, I continued to push the whole day. Later at around 6.30pm, the baby's head could be seen but I did not have any more energy to push. I started to bleed. The doctor examined me and informed (us) that he was referring me to Malindi District Hospital. They called for the ambulance from the District Hospital only to be informed that the vehicle had gone to pick a patient at Chakama and would delay because the connecting road from Chakama to Marafa was in bad condition. Later, we hired a Nissan from Marafa and paid 4000 Kenyan shillings. The labor pains were then very intense. We reached Malindi Hospital at 10pm”*. (23 year-old rural resident with OL and FD)

### Delays in receiving treatment at care facility

Delays experienced at the health care facility contributed to the third delay. These delays included long queues at the facility and doctor unavailability. The women arrived at the hospital with complications, having delayed at home and on the way. The additional delays at the hospital before an intervention was performed added to the first two delays. A woman who had severe PET had to wait for several hours to receive treatment because of a long queue, while another who had OL and FD had to wait for the doctor to be called.

*“We arrived at the hospital around 8pm. I was received well, examined and admitted to the labor ward. But there were so many women waiting to be seen by the doctor. I had to wait until midnight to be given treatment... I am happy about the care I received. My pressure is back to normal”*. (39 year-old rural resident with severe PET)

*“We reached the hospital around 10.00pm. The pains were very intense. I was examined and referred to theatre immediately, but the doctor arrived at around 1.00 am...I waited for so long”*. (23 year-old rural resident with OL and FD)

For some women, travel delays meant that they arrived at the hospital too late, compromising survival of the unborn baby as narrated below.

*“I was in my uncle's house when the pains started at around 11pm. They brought me to Gongoni Dispensary in a Bajaj immediately because I had been advised to deliver in a hospital since I did not have enough blood. When we arrived, I was examined and encourage to walk because the baby was still far. I was in pain the whole night. The next morning they took me to the delivery room, but I could not push any more. At around noon, the doctor called for an ambulance from district hospital, but they were informed that it had gone to pick another patient. I was in pain until around 2 pm, when the ambulance finally arrived. When we reached the District hospital, I was still kept waiting for about 1 hour, because doctor had to be called. The nurses examined me several times, only to tell me later that the baby was dead... I was operated on later to remove the dead baby”*. (18 year-old rural resident with OL)

*“When we arrived at Madunguni Dispensary, the doctor had left. He was called as he lives nearly. He examined me and remarked that the problem was beyond him and that he was referring me to the district hospital. He called for an ambulance. When we arrived here, a nurse examined and gave me some medication. I was bleeding heavily. Later, I delivered but the baby was dead”*. (23 year-old rural resident with APH due to placenta abruption and severe PET)

## Discussion

This paper documents the findings from a study whose main objective was to assess barriers to EmOC services by women who experienced life-threatening obstetric complications or “near miss” in Malindi District, Kenya. Studies investigating barriers to safe motherhood interventions in low-income countries have mainly focused on verbal autopsies and maternal mortality reviews to provide evidence of underlying causes of maternal mortality [27, 28]. However, maternal mortality represents the tip of the iceberg and capturing near-miss morbidity provides a more comprehensive picture of maternal health outcomes [24]. In this study, interviewing women who experienced life threatening obstetric complications before discharge from hospital provided the advantage of obtaining firsthand information, not prone to recall bias from onset of the complication to when the survivors received treatment.

Findings from this study reveal that a combination of delays occurred for the majority of near-miss participants. Many women delayed seeking care, and on reaching the nearest facility, which often was a dispensary or health centre, their conditions necessitated referral to the district hospital. Additional delays occurred at the initial health facility because there were no available ambulances, the lack of regular commuter transport and the long distance to the district hospital, particularly for rural women. When women eventually arrived at the district hospital, there were further delays because of long queues and unavailability of doctors. These findings support the classical three delays framework [5] and are similar to those observed in other resource-limited settings [29–33].

Knowledge about the risks of childbirth and the benefits of skilled attendance should increase preventive care-seeking, while recognition of danger signs and knowledge about available beneficial interventions should increase care-seeking for complications [4, 5]. Although the women in this study generally had good knowledge of the danger signs during pregnancy, this knowledge did not translate into timely care seeking in the advent of a complication. Women who did not experience obstetric complications in previous pregnancies tended to believe that the index pregnancy would also be without problems. Using experience of a previous pregnancy as a risk predicting tool has been reported elsewhere [29, 31]. Birth preparedness, which is an essential part of ANC, not only entails preventive care seeking, but also identification of a health facility with delivery services, making transportation plans and saving money [33]. This practice was not a norm in this setting, partly because a pregnancy was viewed as a normal and natural process, and therefore not planned for. The contribution of financial limitation in hindering access to maternal health services and consequently to the first delay are well documented [4, 16, 29, 32, 34, 35].

While it is expected that women who attend ANC would be more likely to deliver in a health facility [5], we observed that ANC attendance was not linked to a desire to deliver in a health facility. It is however not surprising given that despite high ANC coverage (90 percent) in Kenya, over 50 percent of women are attended by unskilled persons and/or outside a health facility during delivery [17]. While it is acknowledged that multiple factors may influence a woman or families’ choice about whether to deliver at home or health facility [5], planning for facility delivery beforehand and following the plan is likely to influence a positive outcome from the pregnancy. Hypothetically, appropriate care may be provided should a complication arise during delivery, thus preventing the first two delays substantially [33]. The lack of a strong association between ANC attendance and preference to deliver in a health facility has also been observed in rural Uganda [36].

Although women and families may make timely decisions to seek care, they can still experience delays in reaching appropriate care facilities [4, 5]. Delays in reaching appropriate care facilities when women experience life-threatening obstetric complications is reported as a major factor contributing to high maternal mortality and in developing countries [16, 31, 37]. In many situations, access to care for a woman experiencing a life threatening complication is limited to nearby lower level facilities, which are not equipped to handle obstetric complications [5]. In this study we noted that rural women often had to first visit a dispensary or health centre, which meant that they experienced further delays because health workers in these health facilities had to refer them to the district hospital.

Testimonies of rural women in this study also attest to lack of regular commuter transport, long distances and poor road network. Distance is reported to play an important role in the first and second delays because it influences travel time to access EmOC services [5, 16, 31]. Distance plays two roles in influencing use of services; as a discouragement to seek care in the first place and as an actual barrier to reaching a facility after a decision has been made to seek care [5]. An assessment of existence and functionality of EmOC services in this setting revealed geographical inequities in distribution of comprehensive EmOC services with rural women having to travel longer distances to access services [20].

Near miss upon arrival in hospital is an indication of delays in reaching the hospital, either because of a failed referral chain or because women and their caretakers are late in deciding to seek care [38]. These two aspects are strongly evident in our findings. Nevertheless, the findings provide useful insights into the barriers women experiencing life threatening obstetric conditions face in accessing EmOC, which is vital for maternal mortality reduction in Kenya.

The findings confirm a well known need to prioritise the promotion of birth preparedness, by strengthening knowledge of danger signs and skilled birth attendance as a direct responsibility of health services. This study further documents the importance of saving money and making transportation plans during antenatal services or other preventive or health promotion activities as part of birth preparedness. Increasing actual access to EmOC services in underserved areas and/or compensation through an effective referral infrastructure requires a broader involvement of health sector. A broader involvement of other sectors, local administration and communities in overall priority setting will assist in getting consensus and thus support for decreasing all three delays including better roads, communication and emergency transport availability as part of local development. Such broader involvement for health improvement is referred to in a previous paper [20] as part of a multi country study [39] carried out in Malindi district.

### Study limitations

This study had several limitations. First, we only captured information from women who managed to reach hospital. It is quite likely that many women with similar obstetric problems never get to reach hospital and either die at home or survive with serious morbidities. Thus, a comprehensive appraisal of the barriers women with obstetric complications face cannot be provided in this study. Second, although we note delays in obtaining treatment at the health facility as a barrier, these delays are also related to the quality of care and responsiveness of the hospital in treating obstetric emergencies. Quality of care is influenced by multiple health service factors such as availability of supplies, presence of qualified staff, staff attitude, organization of care and efficiency [5]. A comprehensive examination of these factors is beyond the scope of our study.

## Conclusion

The various delays rarely operate in isolation and are likely to be linked. As demonstrated, pregnant women make an attempt to obtain care when an emergency complication occurs. However, delays in making the decision to seek this care, combined with delays in reaching care facilities means that many arrive too ill to be effectively treated. Although factors at the health care facilities contribute to maternal morbidity and mortality, it is important to underscore the role of the first and second delays, which greatly influence the eventual outcome of both mother and unborn baby. Interventions to reduce maternal mortality and morbidity should therefore adequately address pre-hospital barriers in order to have the greatest effect. Findings in this study highlight the need to prioritise and promote birth preparedness, including knowledge of danger signs, identification of skilled care for childbirth, saving money and making transportation plans. Increasing actual access to EmOC services in underserved areas and/or compensation through an effective referral infrastructure that includes adequate good roads, communication and emergency transport is also necessary.
